# Special Issue: 3D Printing for Biomedical Engineering

**DOI:** 10.3390/ma10030243

**Published:** 2017-02-28

**Authors:** Chee Kai Chua, Wai Yee Yeong, Jia An

**Affiliations:** Singapore Centre for 3D Printing, School of Mechanical and Aerospace Engineering, Nanyang Technological University, Singapore 639798, Singapore; wyyeong@ntu.edu.sg (W.Y.Y.); anjia@ntu.edu.sg (J.A.)

**Keywords:** biomanufacturing, biofabrication, bioprinting, 3D printing, rapid prototyping, additive manufacturing, tissue engineering, drug delivery, implants, lab-on-chips

## Abstract

Three-dimensional (3D) printing has a long history of applications in biomedical engineering. The development and expansion of traditional biomedical applications are being advanced and enriched by new printing technologies. New biomedical applications such as bioprinting are highly attractive and trendy. This Special Issue aims to provide readers with a glimpse of the recent profile of 3D printing in biomedical research.

Three dimensional (3D) printing (also known as additive manufacturing or rapid prototyping) originates from a liquid-based stereo-lithography process from the late 1980s [1]. Early development works were mostly around rapid manufacturing (e.g., rapid investment casting [2]) and process optimization (e.g., design of experiments [3]). However, driven by the invention of new printable materials and associated new processes, it is now becoming a highly diversified research field that often demands multidisciplinary efforts. This is evidenced by the penetration of 3D printing into areas that have long been deemed unrelated before, for example water and desalination [4], and shape memory materials [5,6]. For biomedical engineering, 3D printing has a relatively long history of applications, such as biomodels, prostheses, surgical aids, implants, and scaffolds [1]. Recently, a few new bioengineering applications have been emerging; typical examples are tissue/tumor chips [7,8], various forms of bioprinting [9,10,11] and biobots [12,13]. The continued development and expansion of 3D printing in traditional biomedical applications and the emerging new faces of 3D printing motived us to organize this Special Issue, which aims to provide readers with a glimpse of the recent profile of 3D printing in biomedical research.

This Special Issue covers three reviews and nine research articles. In the reviews, Li et al. focus on recent progress in the additive manufacturing of biomimetic constructs [14]. Wang et al. analyze and discuss specifically the bioprinting of hard tissues [15]. Sun et al. present the current status and challenges of engineering the knee meniscus [16]. In the research articles, Lee et al. compared the properties and printability of type A and B gelatin methacryloyl (GelMA) for bioink applications. They discovered that type A GelMA with a high degree of substitution has very good printability at room temperature [17]. Suntornnond et al. developed a simple analytical model for predicting the resolution of the pressure-driven extrusion process, and the experimental data agreed with the model [18]. Wang et al. explored the extrusion of polycaprolactone/graphene composite scaffolds and found that the scaffold hydrophilicity and cell attachment can be significantly improved by adding even a small amount of graphene [19]. Tsai et al. reported another composite by adding magnesium-calcium silicate to polycaprolactone powder, and bone scaffolds were successfully fabricated via a laser sintering method [20]. Shie et al. developed a new photocurable liquid resin which incorporates both polyurethane and hyaluronic acid, and when cultured with mesenchymal stem cells, the 3D-printed hybrid scaffold was able to promote chondrogenic differentiation [21]. Wang et al. reported the printing and clinical use of patient-specific surgical aids in two studies: spinal surgery and complex pelvic fracture [22,23]. Tan et al. reported the development of an elastic biodegradable tube with aligned microfibers, and surprisingly, human mesenchymal stem cells differentiated to smooth muscle lineage on these microfibrous scaffolds in the absence of soluble induction factors [24]. Lastly, Ng et al. showed that polyvinylpyrrolidone-based bioink could play a critical role in improving the cell viability and homogeneity for cells to be printed in droplets [25].
